# Exploring whey and faba bean protein interactions at the oil-water interface: A combined drop tensiometry and microfluidicsstudy

**DOI:** 10.1016/j.crfs.2025.101158

**Published:** 2025-07-28

**Authors:** Dionysios D. Neofytos, Katherine F. Grasberger, Anders Holste, Sandra B. Gregersen, Claus H. Christensen, Milena Corredig

**Affiliations:** a Department of Food Science, Aarhus University, Agro Food Park 48, Aarhus N, 8200, Denmark; b Palsgaard A/S, Palsgaardvej 10, Juelsminde, 7130, Denmark

**Keywords:** Faba bean proteins, Whey proteins, Microfluidics, Protein mixing, Protein adsorption, Emulsifying properties, Oil/water interface

## Abstract

The interfacial properties of model oil–water interfaces stabilized by faba bean protein isolate (FPI), whey protein isolate (WPI), and their mixtures were investigated. Two complementary techniques, microfluidics-based analysis and drop tensiometry were employed to study the impact of protein mixing on interfacial tension, adsorption kinetics, and droplet stability. A microfluidic platform was used for droplet generation and single droplet analysis, assessing the impact of protein blending on droplet size and shape eccentricity after droplet generation. Drop tensiometry complemented the microfluidics-based analysis by evaluating interfacial tension and viscoelastic properties of the various interfaces. The presence of FPI altered WPI interfaces; in mixed systems, antagonistic interactions between proteins resulted in a decreased elastic modulus and broadening of the shape eccentricity. Mixed systems resulted in smaller droplets with narrower size distributions and increased resistance to short-term coalescence with respect to emulsion droplets stabilized by FPI alone, and droplet stability increased proportionally with WPI/FPI ratio.

## Highlights

• WPI and FPI interact at the oil-water interface, forming blends.
• Microfluidics and interfacial tensiometry offer complementary insights, capturing interfacial behavior across timescales.
• Mixed systems exhibited rapid adsorption due to surface-active FPI components.
• Droplet size, distribution, shape eccentricity, and coalescence behavior were influenced by the WPI/FPI ratio.

## Graphical abstract

**Figure:**
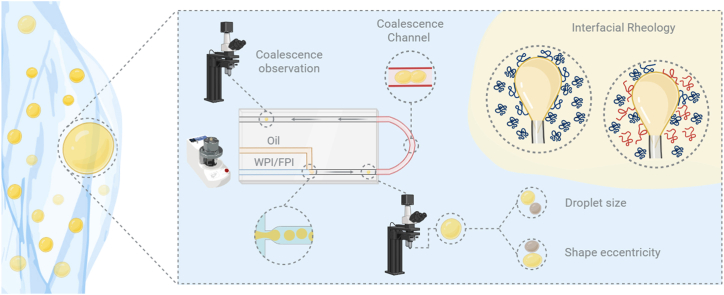

## 1 Introduction

Many food products are complex emulsions. Emulsion formation involves the dispersion of one liquid phase into another immiscible liquid phase. In food, proteins play a crucial role in stabilizing emulsions due to their amphiphilic nature, which allows them to adsorb at the oil-water interface, reducing interfacial tension and forming a protective viscoelastic layer around the droplets. This dynamic process prevents coalescence, where droplets merge to form larger ones, thereby maintaining the stability and uniformity of the emulsion (M. Zhang et al., 2022). Thus, understanding how interfacial layer composition and rheological properties affect droplet size, structure, and resistance to coalescence is crucial for optimizing food product stability and functionality (McClements, 2012).

Among the various emulsifiers studied, particular attention has been given to small molecular weight surfactants and animal-derived proteins due to their well-documented ability to stabilize emulsions. Whey proteins, particularly β-lactoglobulin and α-lactalbumin, are known for their emulsifying properties (Bergfreund et al., 2018; Corredig and Dalgleish, 1995; Woodward et al., 2004). They have been shown to promptly adsorb at the oil–water interface, forming thin, charged viscoelastic interfaces which prevent coalescence. Upon adsorption at the oil–water interface, whey proteins undergo conformational changes which lead to the formation of a cohesive film (Schröder et al., 2017). The structure and viscoelasticity of the final interfacial films depend on protein structure, charge, composition of the bulk phase, and environmental conditions, all of which will ultimately influence emulsion bulk properties (Bergfreund, Bertsch, et al., 2021; Dickinson, 1999; Wilde et al., 2004).

Growing environmental concerns have driven interest in the utilization of sustainable protein sources, including plant-based proteins like pea, soy and faba bean protein isolate as food ingredients. However, the introduction of these ingredients in food formulations has been a challenge, due to their poor solubility, unbalanced amino acid profiles, off-flavors, and overall limited consumer acceptance (Dimina et al., 2022; Vatansever et al., 2024). Combining them with dairy proteins can help overcome some of these challenges, by providing interesting synergisms that could potentially enhance their uptake (Grasberger et al., 2022, 2023; Hinderink, Kaade, et al., 2020).

By taking advantage of the synergistic interactions between different protein sources, mixed systems can be used to achieve acceptable functionality and sensory attributes, while maintaining their sustainable character (Genet et al., 2023). This approach unlocks the ability to rationally develop and optimize plant and dairy protein blends to meet specific formulation requirements in which the unique properties of each protein source can be exploited (Grasberger et al., 2022). However, a detailed understanding of how the individual proteins perform at the interface and what their contribution is in a mixed system is critical to be able to predict their properties. For example, it has been shown that mixed dairy and plant based proteins may affect the interfacial properties of oil droplets, ultimately affecting their stability (Grasberger et al., 2022, 2023; Hinderink, de Ruiter et al., 2021; Hinderink et al., 2019; Hinderink, Sagis, et al., 2020). In this regard, the effect of FPI–whey protein mixed interfaces remain underexplored (Badjona et al.; Martineau-Côté et al., 2022; Shi and Nickerson, 2022; Thomsen et al., 2025).

Studies of emulsions often involve measurements of bulk properties, such as droplet size, viscosity and physical stability. However, this approach does not capture critical details occurring at the nano- and micro-scale. Droplet formation is governed by complex interfacial processes, including emulsifier adsorption, interfacial layer formation and restructuring, and dynamic exchanges between the bulk phase and the interface, all of which contribute to the structural stability required to resist droplet interactions and coalescence (McClements and Gumus, 2016). Therefore, a deeper understanding of these interfacial dynamics is essential to complement and more fully interpret bulk-level observations.

Pendant drop tensiometry enables the characterization of oil–water interface properties by measuring changes in interfacial tension over time, providing insights into emulsifier adsorption dynamics and molecular rearrangements at the interface. The viscoelastic properties of the interface can also be assessed at pseudo equilibrium, by evaluating the response to oscillatory deformations of the droplet volume. However, drop tensiometry provides information over long time scales reflecting the properties of the system at meso-equilibrium (Berry et al., 2015).

During mixing or homogenization, particularly in protein-based systems, the time frames for droplet formation are much shorter (ms scale) and occur in turbulent fields, often followed by multiple stages (Schroën et al., 2020). Hence, it is important to employ complementary techniques to probe interfacial properties under conditions that better reflect the dynamic environments encountered during processing (Olad and Håkansson, 2025). Recent advances in microfluidics have opened new possibilities for studying droplet formation at shorter time scales (Muijlwijk et al., 2017). Microfluidic devices allow for *in situ* observation of emulsification, enabling both the formation and characterization of droplets with a monomodal size distribution (Basu, 2013; Mu et al., 2022) and the study of droplet stability and coalescence under various conditions (Dudek et al., 2020; Hinderink, Kaade, et al., 2020; Mazutis and Griffiths, 2012). Although the laminar flow conditions present in microfluidic devices differ from the turbulent, shear, and convection conditions typically found in industrial homogenization processes, microfluidics-based approaches offer another view point for real-time observation of droplet formation, droplet-droplet interactions, and droplet stability (Ho et al., 2022).

In microfluidic experiments, particularly those using T-junction devices, interfacial tension plays a critical role in determining droplet size during formation. The balance between interfacial tension and shear stress governs the pinch-off dynamics, with lower interfacial tension reducing the energy barrier for droplet breakup and resulting in smaller droplets under equivalent flow conditions. In particular, the addition of emulsifiers, such as proteins or low-molecular-weight surfactants, lowers the oil–water interfacial tension, facilitating earlier necking and faster detachment of smaller droplets. It has been widely acknowledged that interfacial tension is a key determinant of droplet size in such systems, even though droplet stabilization kinetics and surfactant/protein adsorption dynamics also play important roles during and after formation (Abate et al., 2009; Baret, 2012; Garstecki et al., 2006; Glawdel and Ren, 2012; Sohrabi et al., 2020; Wang et al., 2009). Hence, microfluidics enables the systematic observation of single droplets dynamics.

This study focuses on how whey proteins and faba bean proteins adsorb at the oil-water interface and how their mixing behavior, antagonistic or synergistic, may affect droplet formation and short-term coalescence. By leveraging both microfluidic techniques and drop tensiometry, we aim to gain insights into interfacial stabilization at different timescales, probing different properties of the protein mixtures. Unlike previous studies in mixed protein systems, that primarily examine bulk emulsions, in this work we specifically investigated how mixed protein systems may influence *in situ* droplet formation and short-term coalescence. Such insights contribute to a deeper understanding of the emulsification behavior of mixed protein systems.

## 2 Materials and methods

### 2.1 Materials

Whey protein isolate (WPI, Nutrilac BK-9250, Arla Foods Ingredients, Denmark) and faba bean protein isolate (FPI, Tendra® isolate, Cosun Protein, Netherlands) were used as is, per manufacturer's specifications, WPI contained 5.5 % moisture, 92 % protein, 0.2 % carbohydrates, 0.2 % fat and 4.5 % ash, while FPI contained 4.9 % moisture, 84.3 % protein, 3.1 % carbohydrates, 0.3 % fat and 6 % ash. Sodium phosphate (dibasic heptahydrate and monobasic monohydrate), activated magnesium silicate (Florisil®, 100–200 mesh) and 1,4-Dithioerythritol (DTT) were purchased from Sigma-Aldrich (Burlington, MA, United States). Sunflower oil was bought from a local supermarket. MilliQ water was used in all buffers and solutions.

### 2.2 Methods

#### 2.2.1 Oil preparation

To remove any impurities, sunflower oil was stripped using Florisil as described in the literature (Berton et al., 2011; Maldonado-Valderrama et al., 2008). Specifically, the oil was mixed with Florisil at a 3:1 ratio and allowed to interact for 24 h at 4 °C using a rotator mixer. Afterwards, the Florisil/oil mixture was centrifuged at low speed (4700 g) for 30 min (Multifuge 3S-R, Heraeus, Germany). The supernatant was collected and centrifuged again using the same settings to ensure the removal of all Florisil residual. Oil samples were collected and stored at −80 °C, when they were defrosted, they were kept at 4 °C and used within a week. The clean oil-water interface had an interfacial tension of ∼ 28 mN/m.

#### 2.2.2 Protein solutions preparation

Protein solutions were prepared by dispersing the powders in phosphate buffer (10 mM, pH 7.0) and mixing at 45 °C for 30 min at 200 rpm using a Thermomix TM6 (Vorwerk, Germany). The solutions were then kept at 4 °C for 24 h to ensure full hydration. Subsequently, the insoluble material was removed by centrifuging the samples at 15,000 g for 30 min at 4 °C. For the WPI solutions, no pellet was observed after centrifugation, whereas for the FPI solutions, insoluble material formed a pellet, which was discarded. While the chemical composition of the pellet was not further analyzed, previous literature reported the presence of large insoluble protein aggregates, as well as the insoluble carbohydrates in this fraction (Janssen et al., 2024). Since the present study primarily focused on the soluble protein fraction, which directly participates in interfacial adsorption and emulsification, the pellet composition was not further characterized.

The protein content of the soluble fractions was measured using Dumas nitrogen analysis with a Dumatherm (Gerhardt GmbH & Co., Königswinter, Germany), applying a nitrogen factor of 6.38 for whey proteins (Koletzko and Shamir, 2006) and 5.4 for faba bean proteins (Mariotti et al., 2008). Mixtures of a total protein concentration of 1 mg/mL were prepared using the soluble fractions of WPI and FPI, with WPI/FPI protein ratios of 100/0, 75/25, 50/50, 25/75, and 0/100, respectively. All solutions were mixed for at least 30 min at ambient temperature before analysis. Immediately prior to drop tensiometry and microfluidic experiments, the protein solutions were then diluted with phosphate buffer to a total protein concentration of 0.1 mg/mL. Both the oil and protein solutions were filtered through 0.45 μm hydrophobic PTFE and 0.45 μm hydrophilic PVDF membrane filters, respectively (Cobetter Filtration Equipment Co., Ltd).

#### 2.2.3 SDS-PAGE analysis

SDS-PAGE analysis, under both non-reducing and reducing conditions, was conducted on the soluble fraction of all protein solutions using a 4–15 % Criterion™ TGX™ Precast Midi Protein Gel (Bio-Rad, Hercules, USA). For the non-reduced samples, 20 μL of protein sample were mixed with an equal volume of Laemmli sample buffer (20 mM Tris, 2 % SDS, 20 % glycerol, bromophenol blue) to a final protein concentration of 1 mg/mL. For reduced samples, the same procedure was followed with the addition of 20 mM DTT. After mixing, the solutions were heated at 95 °C for 2 min. The gel was then placed in a Criterion Cell (Bio-Rad, Hercules, USA), and the reservoir was filled with Laemmli Running buffer (25 mM Tris, 192 mM glycine, 0.1 % SDS, pH 8.3). 20 μL of each sample were loaded into the wells, resulting in approximately 20 μg of protein per lane. The first well contained PageRuler™ Plus Prestained Protein Ladder, ranging from 10 to 250 kDa (Thermo Scientific™, Massachusetts, USA), serving as a reference. The gel ran for 35 min at 200 V using a Power Pac 200 (Bio-Rad, Hercules, CA, USA), then immersed in a fixing solution (50 % ethanol, 8 % phosphoric acid) for 2 h. To visualize the bands, the gel was stained with Colloidal Coomassie (5 % w/v Coomassie Brilliant Blue in MilliQ water), followed by two washing steps with MilliQ water. Images were captured using the Gel Doc™ EZ System and Image Lab™ software (Bio-Rad Laboratories, USA). Molecular weights were determined via point-to-point regression, and specific polypeptides were estimated based on the literature (Zembyla et al., 2021).

#### 2.2.4 Pendant drop tensiometry

The interfacial tension and dilatational viscoelastic properties of the systems were assessed using a drop tensiometer (Surface Analyzer LSA100, LAUDA Scientific GmbH, Germany). A J-shaped needle with a 1.5 mm diameter was immersed in a 25 mL cuvette containing the protein solution at a concentration of 0.1 mg/mL, and a 30 μL rising drop of stripped sunflower oil was formed. The interfacial tension was then determined (SurfaceMeter™ version 1.2.2.10, LAUDA Scientific GmbH, Germany), based on the Young-Laplace equation, which determines the interfacial tension based on the shape of the created drop, assuming axis-symmetry.

The interfacial tension was measured for >2h (10,800 s) during which a plateau was reached, indicating meso-equilibrium. At this point, the value of interfacial tension ($\gamma_{m}$) was recorded. The interfacial pressure (π_t_ = γ_clean ow_ – γ_t_) was calculated by using a γ_clean ow_ of 28 mN/m and is shown in supplementary information (Fig. S1).

After 10,800 s, the dilatational properties were determined by conducting droplet volume oscillations with amplitudes between 5 and 30 %, with an oscillation frequency of 0.05 Hz. Each volume oscillation was repeated five times with three repetitions and a resting period of 100 s between repetitions. The elastic modulus was determined using the instrument software (SurfaceMeter™ version 1.2.2.10, LAUDA Scientific GmbH, Germany) based on the middle three oscillations and is reported as a weighted average of two independent replicates. To determine the decay time during the adsorption (ꚍ_ad_) and rearrangement (ꚍ_rear_) phases, the interfacial tension isotherms were fitted with a linear decay of two exponentials as shown in equation (1) according to (Grasberger et al., 2024) using MATLAB (MATLAB, Version: 9.13.0.2193358, R2022b). This model provides a phenomenological description of interfacial dynamics, capturing the presence of two dominant kinetic regimes. While the early phase is typically attributed to diffusion- and adsorption-limited transport of proteins to the interface, the slower phase may reflect a combination of conformational rearrangements, protein-protein interactions, and exchanges between the bulk and the interface, including competitive adsorption or desorption effects.

(1) $$\gamma_{t}=\gamma_{\infty }+\left(\gamma_{0}-\gamma_{\infty }\right)\left(a\cdot e^{-\frac{\left(t-t_{0}\right)}{\tau_{ad}}}+\left(1-a\right)\cdot e^{-\frac{\left(t-t_{0}\right)}{\tau_{rear}}}\right)$$

where $\gamma_{t}$, $\gamma_{0}$, and $\gamma_{\infty }$ describe the interfacial tension at a given time t, t = 50 s, and the approximated interfacial tension as the time goes towards infinity, respectively. Parameter *a* is a dimensionless number between 0 and 1, which gives the relative contribution of the two exponentials to the fit function. The decay constants (ꚍ_ad_ and ꚍ_rear_) represent characteristic timescales for the first, faster decay, corresponding mostly to the adsorption of the surfactant and the second portion of the curve, associated with the slower rearrangements/restructuring of the interface, respectively. Corresponding rate constants were calculated by taking the inverse of the decay terms (1/ꚍ_ad_ and 1/ꚍ_rear_).

#### 2.2.5 Microfluidics analysis

**Fig. 1:**
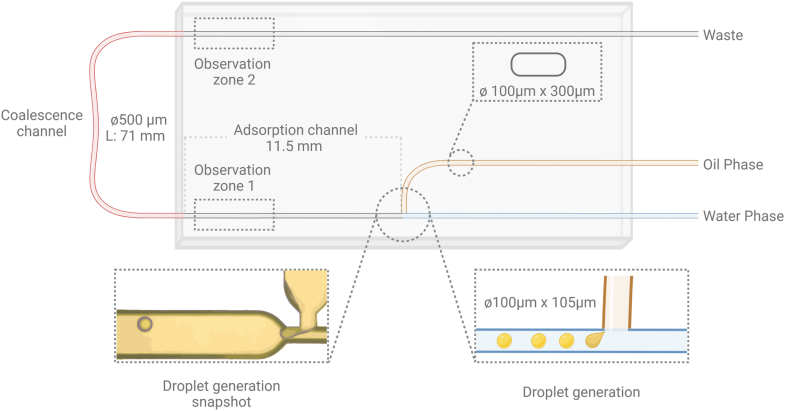
Overview of the microfluidic setup showing the droplet formation zone, coalescence channel, and observation zones 1 and 2.

The emulsification properties of both single and mixed protein solutions, of total concentration of 0.1 mg/mL, were evaluated using a microfluidic platform. This approach assessed the impact of system composition on droplet size, shape eccentricity, and short-term coalescence *in situ.* The microfluidic system generated monodispersed oil droplets within a continuous phase, ensuring control, accuracy, sensitivity, and repeatability. Droplet morphology was analyzed before and after droplets passed through a coalescence channel. A microfluidic setup (Fig. 1) was constructed using commercially available microfluidic components and devices. The studies were performed using a hydrophilic-coated quartz microfluidic chip with a T-junction architecture, channel dimensions of 100 × 300 μm, and a junction size of 100 × 105 μm, provided by Dolomite Microfluidics, UK. High-precision pumps (Dolomite Microfluidics, UK) with flow rate ranges of 70–1500 nL/min for the dispersed phase and 1–50 μL/min for the continuous phase were used to control the flow of both phases. To ensure a stable and consistent flow rate, the pumps were connected to a nitrogen supply instead of compressed air supply.

To obtain oil-in-water droplets, the different protein solutions were placed in the continuous phase reservoir, while the sunflower oil was placed in the dispersed phase reservoir. The flow rates for the dispersed and continuous phases were maintained at 1.5 μL/min and 80 μL/min, respectively, with flow rate deviations of less than 0.05 μL/min for the oil and 1 μL/min for the protein solution. These settings were determined based on preliminary studies (data not shown) on experimental reproducibility.

After formation, the oil droplets traveled through an 11.5 mm adsorption channel, with a residence time of approximately 0.259 s, assuming that the droplets travel with the fluid's average velocity. This residence time allowed proteins to adsorb to the interface before entering the coalescence channel. Following the adsorption channel, the droplets traveled through a PTFE tube, termed as “coalescence channel”, with a diameter of 500 μm and a length of 71 mm with residence time of approximately 10.45 s, assuming that the droplets traveled with the fluid's average velocity. PTFE was chosen as the most inert material available due to its low surface energy, inherent hydrophobic behavior, non-porous surface and non-reactive nature. As the droplets entered this channel, the expansion in channel dimensions resulted in a decreased average velocity of the fluid (water phase). This lower continuous phase velocity allowed the droplets to interact with each other, providing an opportunity to observe their stability against short-term coalescence (Muijlwijk et al., 2017). The coalescence channel was wider than the observation zones to facilitate droplet interactions and potential coalescence; this narrowing at the end of the channel may introduce shear forces on the droplets. Although, as all samples were subjected to the same flow conditions, these effects were consistent across all tested systems, ensuring comparability.

Immediately after droplet formation and after passing through the coalescence channel (Fig. 1, observation zones 1 and 2, respectively), a video consisting of 200 frames at 152 frames per second (FPS) was captured using a high-speed microscope camera (Meros High-Speed Digital Microscope, Dolomite Microfluidics, UK). Observation zone 2 was kept narrower than the coalescence channel to maintain a stable focal plane for imaging, minimizing positional variability and enabling precise droplet tracking and shape analysis. Droplet morphometry analysis was conducted on the captured videos using Droplet Morphometry and Velocimetry (DMV) software (Basu, 2013) to monitor both the equivalent diameter and shape eccentricity of droplets before and after they traveled through the coalescence channel.

The droplet equivalent diameter represents the diameter of a perfect sphere with the same mass, volume, or area as the observed droplet (Basu, 2013; Piacentini, 2015). Shape eccentricity describes the degree to which an object deviates from a perfect circle, ranging from 0 to 1, with values close to 1 indicating a more elongated shape and values close to 0 a more spherical shape (Basu, 2013; Lubarda and Talke, 2011). In this study, eccentricity was used as an indicator of the droplet's ability to maintain a spherical form. Higher eccentricity values suggest interfacial instability or delayed interfacial relaxation, likely driven by protein interactions and variations in interfacial film strength.

For all protein systems, droplet analysis was conducted on ∼ 200 droplets for each of three replicates (totaling ∼ 600 droplets). The distribution of both the equivalent diameter and shape eccentricity in relation to their frequency were visually represented as histograms, and a Gaussian distribution was fitted to the histogram data to obtain the mean droplet size and standard deviation of each of the independent measurement/replicate and for each WPI/FPI ratio. The number and width of the bins used in the histogram were selected using the Freedman–Diaconis rule (Freedman and Diaconis, 1981). Although individual droplets were analyzed in flow with the aim of capturing unique droplets, the possibility of occasional re-capture of the same droplet across successive frames cannot be fully excluded. However, given the sample size derived from three independent biological replicates per system, and the consistent application of identical analysis conditions across all systems, the statistical robustness and validity of the results were considered to be unaffected by this possibility.

### 2.3 Statistical analysis

All microfluidics experiments were performed in triplicate, showing reproducibility. Drop tensiometry data were performed in duplicate, with three subsamples to take into account for instrumental variation. All data presented in the oscillating drop tensiometry and microfluidics-based droplet analysis sections represent weighted average values, calculated by incorporating the standard deviation of each independent measurement into the mean value using the following equations:

(2) $$\bar{x}=\frac{\sum_{i=1}^{n}w_{i}x_{i}}{\sum_{i=1}^{n}w_{i}}$$

(3) $$\bar{\sigma }=\sqrt{\frac{1}{\sum_{i=1}^{n}w_{i}}}$$

(4) $${\;w}_{i}=\frac{1}{{\sigma_{i}}^{2}}$$

where $\bar{x}$ the weighted average, n the number of terms, $w_{i}$ the weight applied to each $x_{i}$ value, $\sigma_{i}$ standard deviation of each measurement and $\bar{\sigma }$ the combined standard deviation, representing the overall uncertainty of the weighted average.

For the analysis of equivalent diameter and shape eccentricity, a one-way ANOVA was conducted for all samples. The resulting p-value for the F-statistic was below 0.05, indicating that at least one treatment was significantly different from the others.

Post-hoc Tukey HSD tests further confirmed that all sample pairs exhibited statistically significant differences, except for the shape eccentricity data of the 75/25 and 0/100 (WPI/FPI) mixtures, where no significant difference was observed. The results of the Tukey HSD tests are provided in the supplementary information (Table S1).

## 3 Results & discussion

### 3.1 Protein profile characterization

**Fig. 2:**
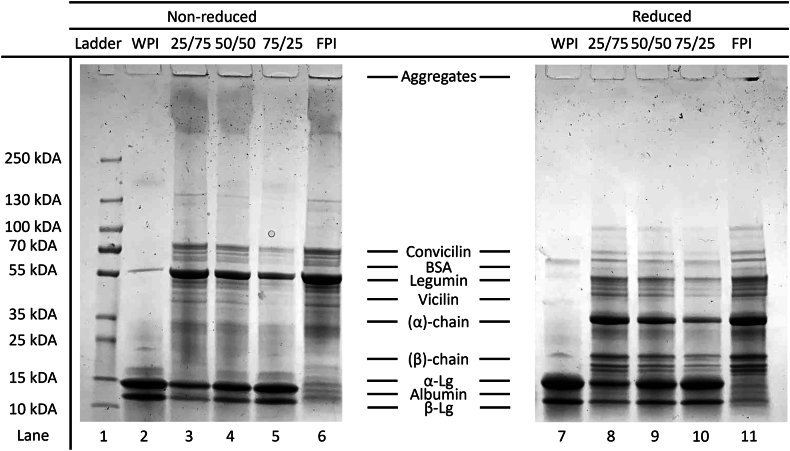
SDS-PAGE of the protein dispersions analyzed under non-reducing (lanes 2–6) and reducing (lanes 7–11) conditions: WPI (lanes 2 and 7), FPI (lanes 6 and 11), mixed systems with ratios of 25/75 (lanes 3 and 8), 50/50 (lanes 4 and 9), 75/25 of WPI/FPI (lanes 5 and 10) and molecular weights of the protein ladder (Lane 1). Bands are labelled according to literature (Badjona et al.; Madureira et al., 2007).

Fig. 2 shows the protein electrophoretic profile for the protein solutions used to prepare the samples.

The protein composition of WPI under non-reducing conditions (Fig. 2, lane 2) predominantly comprised β-lactoglobulin (∼ 18 kDa), α-lactalbumin (∼ 14 kDa), and a minor band around ∼ 60 kDa, corresponding to BSA, consistent with typical bovine WPI composition (Madureira et al., 2007). Under non-reducing conditions, the FPI (Fig. 2, lane 6) showed prominent bands for legumin (∼ 55 kDa), vicilin (∼ 45–55 kDa), and, in smaller amounts, convicilin (∼ 70 kDa) and albumins (<15 kDa). These bands were identified based on previous literature (Vogelsang-O’Dwyer et al., 2020; Żmudziński et al., 2021). Under reducing conditions, the reduction of disulfide bonds within legumin resulted in the dissociation of its subunits, as evidenced by a decrease in the intensity of the ∼ 55 kDa band (lane 11) and an increase in bands corresponding to the two legumin subunits (α-chain and β-chain) at their respective sizes of ∼ 35 kDa and ∼ 20 kDa. Overall, the protein profile of FPI was consistent with previous findings (Singhal et al., 2016; Vogelsang-O’Dwyer et al., 2020). In the case of the non-reducing gel, the upper part of the lane for both single FPI and WPI/FPI mixed systems (lanes 3–6), present a small protein clumping in the well as well as longitudinal smearing across the lane, indicating the presence of large protein aggregates in the samples. The smearing was more intense in the case of FPI while in the case of mixed systems it became less noticeable with increasing WPI ratio. Both the smearing and the intensity of the high molecular weight bands at the top of the lanes in FPI and WPI/FPI mixed samples were not visible under reducing conditions (lanes 8–11), indicating the presence of covalently linked aggregates. It is important to note that even under non-reducing conditions, the legumin's α-chain and β-chain were present in a dissociated form, albeit at lower intensity (lane 6).

### 3.2 Adsorption dynamics

#### 3.2.1 Interfacial tension

**Fig. 3:**
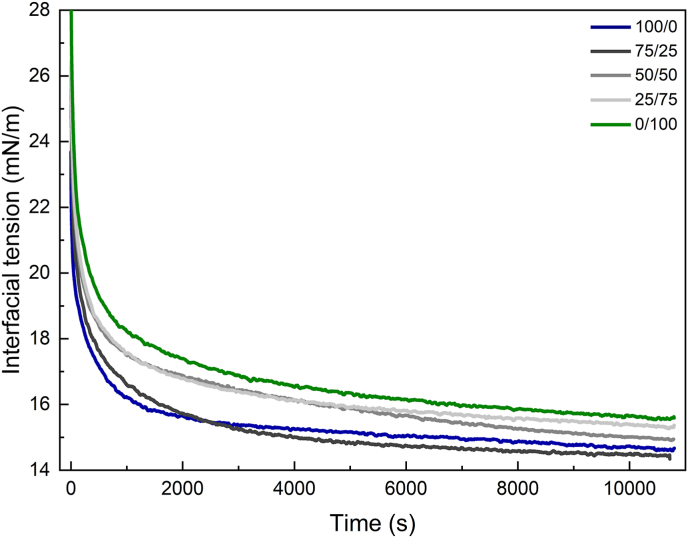
Interfacial tension isotherms of the sunflower oil-water interface with 0.1 mg/mL of total protein for the WPI/FPI mixtures of: 100/0 (blue), 75/25 (black), 50/50 (dark grey), 25/75 (light grey) and 0/100 (green).

Pendant drop tensiometry was employed to study the adsorption dynamics of both single and mixed protein systems at the oil-water interface. As shown in Fig. 3, the interfacial tension of all protein-stabilized systems decreased rapidly during the initial phase (<1000 s), followed by a slower rate of decrease thereafter. The corresponding interfacial pressure increase is shown in supplementary information (Fig. S1). After the initial fast decrease in interfacial tension, the slower decrease corresponds to further reactions, such as proteins continuing to adsorb, interfacial exchanges between the bulk and the interface, displacements and rearrangements at the interface, eventually reaching meso-equilibrium, as indicated by the plateau region where complete saturation of proteins at the interface was achieved.

All protein solutions were tested at the same concentration to facilitate the comparison between the samples. The WPI-stabilized interface reached a plateau at 15.0 mN/m ± 0.3, aligning with previous reports (Grasberger et al., 2022). Table 1 presents the interfacial tension ($\gamma_{m}$) and surface pressure ($\pi_{m}$) values at 10,800 s. The interfacial tension value for the WPI-stabilized interface was lower than that for the FPI-stabilized interface, which exhibited the highest $\gamma_{m}$ among all protein treatments (15.5 mN/m ± 0.1). The mixed protein systems showed intermediate values (Table 1), with $\gamma_{m}$ decreasing as the WPI/FPI ratio increased. Up to a 50/50 ratio, the $\gamma_{m}$ of the mixed systems appeared to be dominated by FPI. In the mixture containing 75 % (w/w ratio) whey protein, the system exhibited comparable values, but lower, to that of WPI.

**Table 1:** Average interfacial tension and pressure values for single and mixed protein interfaces measured after reaching plateau at 10,800 s.

Interface Composition (WPI/FPI)	$\gamma_{m}$ (mN/m)	$\pi_{m}$ (mN/m)
100/0	15.0 ± 0.3	13.0 ± 0.3
75/25	14.5 ± 0.1	13.5 ± 0.1
50/50	15.2 ± 0.2	12.8 ± 0.2
25/75	15.5 ± 0.1	12.5 ± 0.1
0/100	15.5 ± 0.1	12.5 ± 0.1

Overall, the interfacial tension was inversely proportional to the WPI/FPI ratio. It should be noted that even though the protein weight ratios were set at 75/25, 50/50, and 25/75, the actual molar ratio of the proteins did not reflect the same relationship, as WPI proteins are smaller (i.e. 18 kDa for β-lactoglobulin monomer) than FPI proteins (several hundreds of kDa).

#### 3.2.2 Adsorption kinetics

To further understand potential differences in the kinetics of adsorption, the decay of the interfacial tension was compared between treatments, based on the analysis of the two overlapping regions of the interfacial isotherms (Equation (1)). The inverse of the decay times, representing the rate of decrease in interfacial tension, are reported in Table 2. Although the adsorption and rearrangement may overlap, the two phases reflect the two dominant mechanisms, first adsorption and then, rearrangement, respectively, and are therefore referred to as the adsorption and rearrangement rates. The rate of adsorption was found to be faster for FPI compared to WPI. Within FPI, specific protein fractions such as vicilin (∼ 130–150 kDa) and albumins (<15 kDa) are the main proteins dominating the initial adsorption due to their solubility and surface activity, whereas in WPI, β-lactoglobulin (∼ 18 kDa) and α-lactalbumin (∼ 14 kDa) govern interfacial behavior (Vogelsang-O’Dwyer et al., 2020; Żmudziński et al., 2021). These results align with previous reports for another plant protein extract, commercial pea protein isolate, which also showed faster adsorption kinetics compared to whey proteins (Singhal et al., 2016); however, it is important to note that the heterogeneous composition of the plant protein fractions may play an important role, with the most surface active components reaching the interface first, with other components being more slowly adsorbed. The presence of lower molecular weight components, such as peptides or other small non-protein compounds, could enhance initial adsorption. In particular, plant protein isolates, including FPI, may contain surface active compounds such as phenols, saponins, and phospholipids, which have been shown to contribute to adsorption dynamics (Arora and Damodaran, 2011; Bandyopadhyay et al., 2025; Kamani et al., 2024; Keuleyan et al., 2025; Sawicki et al., 2024; Singh et al., 2017; Stone et al., 2024; Zhu et al., 2019).

**Table 2:** Adsorption and rearrangement rate of both single and mixed protein interfaces.

Interface Composition (WPI/FPI)	k_ads_ X 10^−4^ (s^−1^)	k_rearr_ X 10^−4^ (s^−1^)
100/0	24.0 ± 3.1	1.6 ± 0.6
75/25	28.2 ± 1.7	4.1 ± 0.3
50/50	29.6 ± 4.4	1.8 ± 0.1
25/75	33.1 ± 0.3	2.6 ± 0.2
0/100	31.0 ± 2.7	2.3 ± 0.4

The globular nature of the proteins in both FPI and WPI suggests that initially, the hydrophobic regions may be less exposed, with rearrangements occurring at longer timescales. This has been shown, for example, for β-lactoglobulin which, after the initial adsorption, undergoes rearrangements driven by the exposure of its hydrophobic core to the oil phase, leading to a robust interfacial protein network via intermolecular disulfide bonds (Benjamin et al., 2014; Bergfreund et al., 2018; Dickinson and Matsumura, 1991; Wu et al., 2011, 2021). This is consistent with the slow rearrangement rate and high elastic modulus observed for WPI.

Table 2 also shows the differences in rearrangement rates between the treatments. The rearrangement rate of FPI was faster than that of WPI. Despite being an industrially processed isolate, FPI's rearrangement rates were similar to those reported for mildly processed pea proteins (Grasberger et al., 2024). This contrasts with reports indicating that industrially processed plant protein isolates exhibit limited structural reorganization upon interfacial adsorption. This behavior is typically attributed to their harsh processing history, which leads to extensive denaturation, aggregation, and a loss of molecular flexibility, resulting in structurally compromised proteins that are less capable of undergoing conformational rearrangement at interfaces (Hinderink, Berton-Carabin et al., 2021; Ma et al., 2022; Tang et al., 2009; H. Zhang et al., 2022). While the speed of the rearrangement rate can indicate the extent of network formation (i.e., slow rearrangement, increased network formation), this is not always the case, especially for mixed systems. In these systems, competition due to the different adsorption dynamics of the surface-active components may result in increased interfacial rearrangements due to displacement, causing longer term rearrangements in bulk emulsions. Therefore, it is important to evaluate such rearrangement rates, together with the viscoelastic properties at the interface measured at meso-equilibrium, to better predict interfacial properties in mixed protein systems.

#### 3.2.3 Interfacial viscoelastic properties

**Fig. 4:**
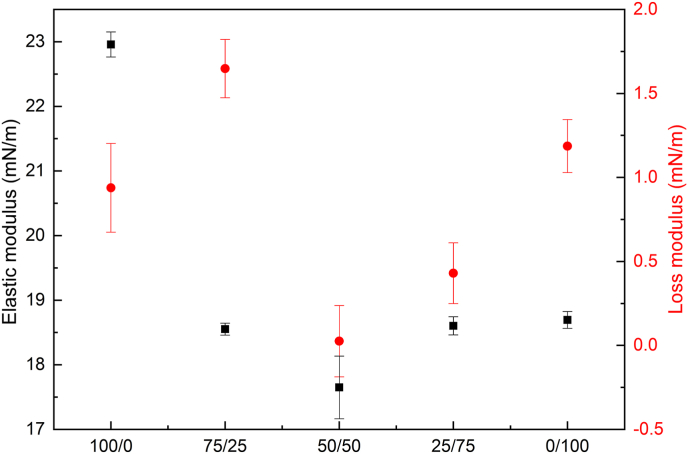
Elastic modulus (black squares) and loss modulus (red circles) of various protein solutions at 5 % volumetric oscillations, based on their weight ratios, for both single and mixed (WPI/FPI) systems: Each value represents the weighted average of two independent replicates, with error bars indicating the combined standard deviation, reflecting the overall uncertainty of the weighted average.

Amplitude sweeps were conducted up to 30 % deformation to assess the interfacial response under increasing perturbations (Figs. S2 and S3). However, as the focus of this study was on the behavior at small deformations (5 %), Fig. 4 summarizes the elastic modulus measured at 5 % amplitude deformation. It is important to note that these viscoelastic data were measured after the plateau of interfacial tension was reached and therefore may not fully reflect the viscoelastic properties at the initial stages of adsorption. The viscous modulus (Fig. 4) was much lower in all systems (<2 mN/m). WPI showed the highest elastic modulus, while interfaces containing FPI showed a significantly lower value (Fig. 4), indicating a less stiff network. All blends showed fast adsorption rates like those of the 100 % FPI-stabilized interface (Table 2), suggesting that in the mixed systems, faba protein isolate components dominated the initial adsorption phase. The observed similarity in rearrangement rates, with the exception of the 75/25 WPI/FPI blend, suggests that the faba proteins, when present in sufficient amount, may hinder the optimal adsorption and rearrangement of whey proteins. In WPI-stabilized interfaces, β-lactoglobulin (∼ 18 kDa) and α-lactalbumin (∼ 14 kDa) rapidly adsorb and undergo conformational changes, leading to the formation of a cohesive and elastic interfacial film. In contrast, FPI contains larger globular proteins such as vicilin (trimer of ∼ 130–150 kDa) and legumin (hexamer of ∼ 300–380 kDa), which adsorb differently due to their oligomeric structures and polydispersity. These proteins may have a lower ability to form strong intermolecular interactions at the interface, leading to less efficient packing and a more heterogeneous network (Benjamin et al., 2014; Bergfreund, Bertsch, et al., 2021; Bergfreund et al., 2018; Bergfreund, Diener, et al., 2021), potentially explaining the decreased elastic modulus.

The rearrangement rates at the highest WPI/FPI ratios (75/25) were faster than for any other mixture. Considering that this blend also reached the lowest interfacial tension, it may be assumed that the main network was formed by WPI, and only partially disrupted by FPI components. The presence of FPI however, still sufficiently decreased the interfacial dilatational elasticity modulus. These results highlight the complex interplay between protein ratio, interfacial tension, and network formation.

Overall, regardless of the ratio, the different adsorption and rearrangement kinetics of faba and whey proteins, when combined, suggest an antagonistic effect on interfacial network formation. This antagonistic effect has been reported in previous studies on protein mixtures or proteins/surfactant mixtures, where competitive adsorption at the interface can disrupt optimal network formation (Ho et al., 2018; Rippner Blomqvist et al., 2004; Zhou et al., 2022).

It is clear from drop tensiometry data that the presence of FPI affected the interfacial structure and showed less elastic interfaces. However, the measurements were carried out at much longer timescales than those relevant during droplet formation, and therefore microfluidics was also employed to compare the protein systems.

### 3.3 Droplet formation and coalescence observations using microfluidics

Microfluidic observations revealed dynamic interfacial rearrangements, including droplet elongation, delayed coalescence, and interfacial deformation, which were not captured by equilibrium interfacial tension measurements. These real-time observations provided additional insights into the stabilization mechanisms of the protein solutions studied.

When employing microfluidic devices for droplet generation, four main factors significantly impact droplet size: (1) the flow rates of the continuous and dispersed phases, (2) the microfluidic chip architecture, (3) the viscosity of the different phases, and (4) the interfacial properties of the systems under analysis (Sartipzadeh et al., 2020; Srikanth et al., 2021). For comparison reasons, during microfluidics experiments the concentration of the protein solutions was 0.1 mg/mL, this concentration was equivalent to that used in the drop tensiometry experiments. At this concentration there were no differences in the continuous phase viscosity between the samples. Furthermore, low and consistent flow rates were employed for both dispersed and continuous phases to better identify the impact of the protein type and ratios on droplet morphology. The time scales of the microfluidic experiments are significantly shorter than those of drop tensiometry experiments, but the low flow rates ensured laminar flow during droplet formation, limiting the likelihood of turbulent flow and mixing effects, which can obscure the underlying relationships between continuous phase composition and droplet morphology. At the same time, low flow rates allowed more time for interactions and rearrangements between the dispersed and continuous phases; however, it is important to acknowledge that the time scales of the microfluidic experiments are significantly shorter than those of drop tensiometry experiments.

With the above considerations, it can be presumed that differences in droplet size and morphology will occur only due to differences in the way the protein solutions form and stabilize the oil/water interface (Xu et al., 2012).

#### 3.3.1 Effect of protein mixing on droplet size and distribution

**Fig. 5:**
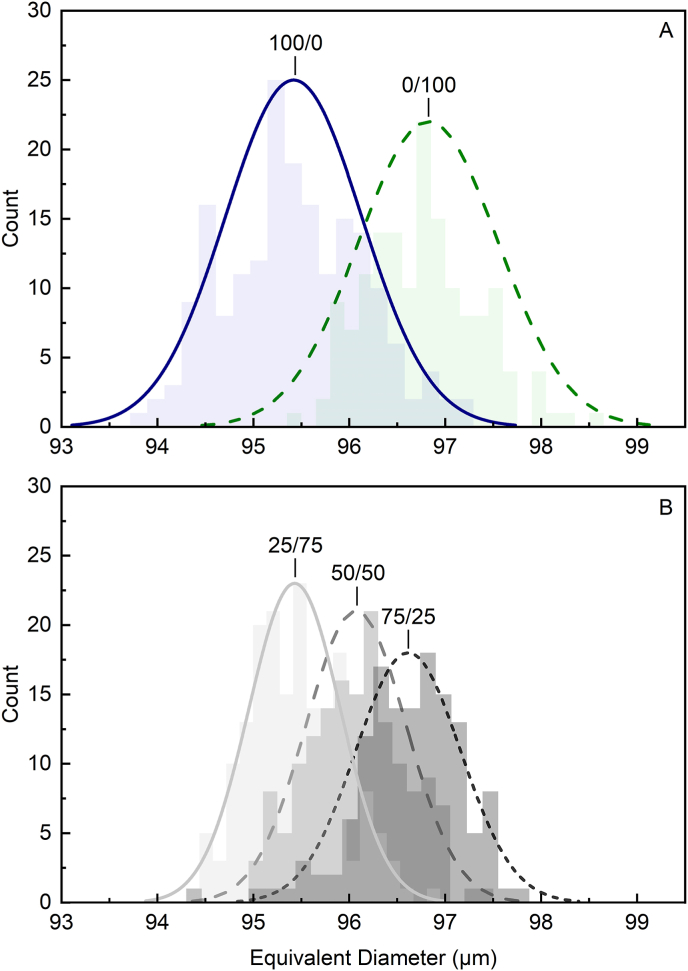
(A) Representative equivalent diameter histogram and Gaussian fitted peaks for droplets stabilized by 0.1 mg/mL WPI (solid line, blue) and FPI (dashed line, green) solutions. (B) Representative equivalent diameter histogram and Gaussian fitted peaks for emulsion droplets stabilized by WPI/FPI mixtures at different protein ratios, immediately after droplet formation (observation zone 1, Fig. 1). The short-dashed line (dark grey) represents a ratio (WPI/FPI) of 75/25, the dashed line (medium grey) represents a ratio of 50/50, and the solid line (light grey) represents a protein ratio of 25/75.

Fig. 5 shows the equivalent diameter of the oil droplets formed immediately after droplet formation in the microfluidic chip (measured in the observation zone 1, Fig. 1) and illustrates the droplet size distribution, expressed by the droplets' equivalent diameter, along with the fitted Gaussian distribution for emulsion droplets stabilized using both single (Fig. 5A) and mixed (Fig. 5B) protein systems.

All generated droplets were within the expected size range, based on the chip design and flow rates. Nevertheless, variations in droplet size were observed because of the presence of different proteins at the interface (Fig. 5). WPI-stabilized droplets revealed a mean diameter of 94.8 ± 0.3 μm (Table 3) and a value of 1.6 for the width of the peak at the point that the full width of the peak is at half of its maximum (1/2_max_). At the same time, FPI-stabilized droplets had a slight shift to larger mean diameters, with a value of 96.5 ± 0.5 μm with a slightly larger width, at 1.8. As the standard deviation of the means can provide information regarding variability in droplet sizes within the sample and the width can reveal differences in the distribution of droplet sizes, these values were compared in Table 3. FPI-stabilized droplets also showed a wider range of diameters, and a less uniform size distribution compared to the WPI-stabilized droplets. However, in both cases, the emulsions were well formed and with comparable size ranges.

**Table 3:** Mean weighted equivalent diameter and weighted average full width at ½ max of the fitted Gaussian peaks for both single-protein and mixed WPI/FPI-stabilized droplets. Data are presented as weighted means across three independent replicates, with the corresponding combined standard deviations.

Interface composition (WPI/FPI)	Weighted equivalent diameter	Combined standard deviation	Weighted average full width at ½ max	Combined standard deviation
100/0	94.8	0.3	1.6	0.1
75/25	96.4	0.3	1.0	0.1
50/50	95.9	0.4	1.2	0.1
25/75	95.2	0.3	1.2	0.1
0/100	96.5	0.5	1.8	0.1

The adsorption dynamics significantly influence droplet formation and interfacial properties (Kovalchuk et al., 2018). Thus, the observed variations among samples, including both droplet size and size distribution, can be largely attributed to the different protein properties. The observed trends among the samples using microfluidics corresponded well to the trends observed among the samples for both interfacial tension as well as the adsorption rates measured by drop tensiometry. The smaller average diameter for WPI-stabilized droplets compared to FPI-stabilized droplets was well aligned with the lower interfacial tension obtained for WPI. It is important to note that in microfluidic systems, the protein solution flows continuously over surfaces, potentially leading to different adsorption mechanisms compared to static methods like pendant drop tensiometry, where protein molecules diffuse mainly due to Brownian motion. The continuous flow in microfluidics, especially with the large FPI proteins, may likely result in a higher number of collisions with the proteins at the interface, possibly affecting the protein adsorption, composition, and rearrangement at the oil/water interface in the mixed systems. Although the mean droplet diameter of the WPI/FPI blends (Fig. 5B) showed average values, in between those measured for the two single protein systems, there was an increase in the droplet size diameter with an increasing WPI/FPI ratio. The blends also showed a narrower size distribution compared to those of the two WPI and FPI systems. These results are in line with the drop tensiometry data shown in Table 2, where protein rearrangement rates were higher in mixed systems compared to the single systems, even if the timescale of the two measurements are very different. This suggests that with mixed protein systems, adsorption, exchange of components and their rearrangements will continue to occur at the interface, providing synergies which result in a sharper gaussian distribution of oil droplets during emulsification by microfluidics. In this system, interfacial tension effects and viscoelastic effects will occur simultaneously and cannot be distinguished.

In the mixed systems, the lowest average droplet diameter was observed at the lowest WPI/FPI ratio and the largest with 75/25 WPI/FPI, indicating that a small amount of WPI could enhance the emulsifying behavior of FPI. By increasing the WPI ratio, the competition of protein molecules for a place at the interface may result in less efficient interface stabilization, thus larger droplets. This observation highlights the interplay between protein composition and interfacial dynamics, emphasizing the importance of considering both equilibrium and dynamic adsorption processes when designing protein-based emulsions.

#### 3.3.2 Effect of protein mixing on droplet shape eccentricity

**Fig. 6:**
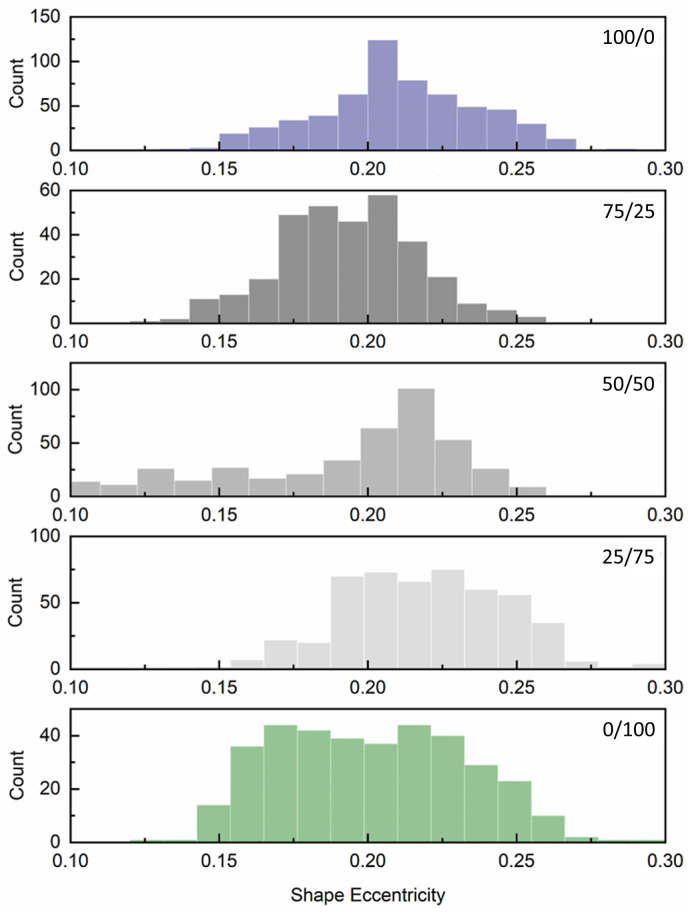
Representative stacked histograms showing the distribution of droplet shape eccentricity values in observation zone 1 (immediately after formation), for emulsions stabilized by whey protein isolate (WPI) and faba bean protein isolate (FPI) mixtures at WPI/FPI ratios of 100/0, 75/25, 50/50, 25/75, and 0/100.

The shape eccentricity of the droplets was also observed during emulsification. The changes in this value, which ranges from 0 to 1, with 0 indicating a perfect spherical shape, are summarized in Fig. 6. As droplets move within the microchannel, interactions between the interface and the moving fluid phase (water) can exert forces that lead to droplet deformation. For instance, shear forces can cause droplets to stretch or elongate (Che et al., 2018). In this study, since the water flow was kept consistent across samples, shape eccentricity was primarily influenced by changes in (1) the interfacial tension, (2) the rate and extent of protein adsorption at the droplet interface, and (3) the shear and dilatational properties of the interfacial layer. Hence, changes in shape eccentricity immediately after droplet generation are interpreted as reflecting the net effect of protein adsorption on both interfacial tension and interfacial mechanical properties (shear and dilatational elasticity), which may act in opposing directions on droplet deformation. In particular, synergistic or antagonistic interactions between proteins at the interface can influence this balance ultimately affecting the observed droplet shape as the newly formed droplets move through the continuous phase. It needs to be mentioned that the observed shape eccentricity values are not the result of improper fusion or coalescence of droplets, as droplets were analyzed individually in flow, shortly after generation, before entering in the coalescence channel. Shape eccentricity was considered a good indicator of interfacial stability and the underlying physicochemical dynamics occurring immediately after emulsification; in other words, increased eccentricity coefficients would reflect suboptimal interfacial film formation.

WPI-stabilized droplets (Fig. 6A) showed slightly lower eccentricity values compared to FPI-stabilized droplets, which had higher eccentricity values. The differences in the histogram distributions suggest that the FPI-stabilized droplets had a broader shape eccentricity range compared to the WPI-stabilized ones. This could be attributed to the differences in adsorption kinetics, elastic modulus, interfacial packing density and compositional heterogeneities, when comparing WPI with FPI containing interfaces. Furthermore, this highlights the contrast between equilibrium interfacial properties observed in tensiometry and the dynamic adsorption and competition processes occurring during droplet formation in microfluidics, where rapid interfacial rearrangements may influence stability and structure. At the short timescales relevant to microfluidics, FPI appeared unable to form a coherent interfacial viscoelastic network, which may have contributed to a broader shape eccentricity distribution due to interface inhomogeneity. For WPI/FPI blends, shape eccentricity values were closer to those observed for WPI-stabilized droplets, particularly in the 25/75 (WPI/FPI) system (Fig. 6D). However, as the WPI content increased (50/50 and 75/25, WPI/FPI), droplet eccentricity showed a decreasing trend (Fig. 6C and B). In the 50/50 (WPI/FPI) mixed system, droplet shape eccentricity exhibited the widest distribution among the samples, indicating greater variability in droplet deformation. This broader distribution suggests increased interfacial compositional heterogeneity, likely due to competition between WPI and FPI at the interface. This antagonistic interaction led to incoherent protein adsorption and network formation, resulting in a wide eccentricity distribution.

Post-hoc Tukey HSD tests further confirmed that all sample pairs exhibited statistically significant differences, except for the shape eccentricity data (Table 4) of the 75/25 and 0/100 (WPI/FPI) mixtures, where no significant difference was observed. The shape eccentricity data aligned well with the drop tensiometry analysis, where FPI stabilized systems showed faster adsorption compared to WPI interfaces. WPI-stabilized droplets had a higher eccentricity value, in other words, they had a more elongated shape compared to the droplets stabilized by FPI. WPI-stabilized interfaces showed a higher elastic dilatational modulus, compared to FPI.

**Table 4:** Mean weighted shape eccentricity values for both single and mixed WPI/FPI stabilized droplets, measured after entry into observation zone 1, as illustrated in Fig. 1. Data are presented as weighted means across three independent replicates, with the corresponding combined standard deviations.

Interface Composition (WPI/FPI)	Weighted shape eccentricity	Combined standard deviation
100/0	0.23	0.01
75/25	0.20	0.02
50/50	0.19	0.02
25/75	0.24	0.01
0/100	0.25	0.01

Emulsions containing 25/75 (WPI/FPI) not only showed the lowest average diameter than all the other mixes but showed the largest shape eccentricity among the mixed systems, suggesting increased rearrangements due to the interactions between WPI and FPI proteins. These results indicate that during droplet formation, interactions between the smaller whey proteins and faba proteins led to the formation of an interface containing faba proteins but where whey proteins still formed a functional viscoelastic layer. The rearrangement values for the 25/75 system were between those of the WPI and FPI stock systems, indicating dynamic rearrangement of proteins from both WPI and FPI at the interface to minimize system free energy. As the WPI protein ratio increased to 50 % and 75 % (50/50 and 75/25, WPI/FPI respectively), both adsorption rate, rearrangement rate, and shape eccentricity decreased while droplet size increased. This suggests that increased competition for interface adsorption among the proteins (Dickinson, 2011; Grasberger et al., 2022, 2023 Hinderink et al., 2022) led to a less stable interface during droplet formation, resulting in larger droplets.

The lower shape eccentricity values under these conditions (50/50 and 75/25, WPI/FPI respectively) suggest that increasing the WPI ratio reduced the competition between proteins, leading to a better-formed protein network, and more spherical droplets. However, the average diameter data would also suggest that the residual FPI affected the viscoelastic properties of the interface. Overall, the shape eccentricity data aligned well with the drop tensiometry observations, indicating that the blends are less stable due to competitive displacement and interactions between the proteins, with consequences to the properties at the interface. It is important to note that these effects were clearly observed within the short timescales used during emulsification by microfluidics.

#### 3.3.3 Effect of protein mixing on droplets short-term stability

The short-term stability of protein-stabilized droplets in a microfluidic system was investigated by monitoring their shape and size after passing through the coalescence channel (observation zone 2, Fig. 1) compared to their size before entering the channel (observation zone 1, Fig. 1). Additionally, to quantify the extent of short-term coalescence among the samples, the proportion of droplets exiting the coalescence channel with the same equivalent diameter as those entering it was estimated for each sample and expressed as a percentage, termed as stability ratio (SR). Consequently, a system with no coalescence was considered to have an SR of 100 %, while a system with no stability at all was considered to have an SR of 0 %.

**Fig. 7:**
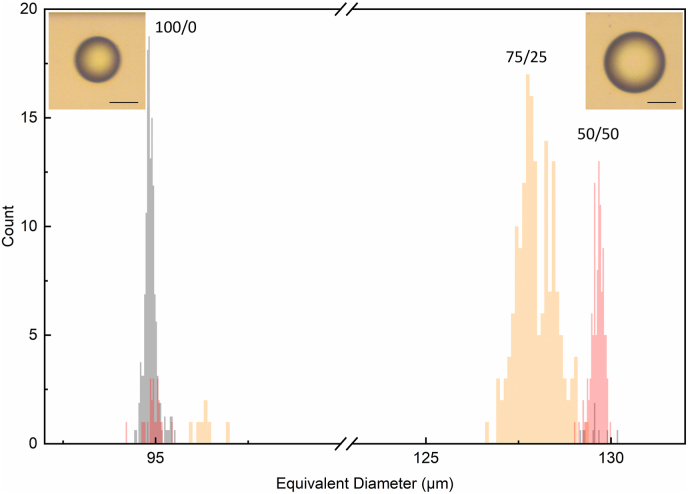
Droplet equivalent diameter of the droplets after the coalescence channel transit (observation zone 2, Fig. 1). Samples prepared with WPI (grey) and 50/50 (red), 75/25 (orange) WPI/FPI hybrid systems. Inset shows representative microscopic images of small (∼ 95 μm, left) and large (∼ 130 μm, right) droplets stabilized by the 50/50 WPI/FPI mixed system, before and after passing through the coalescence channel. Scale bar, 60 μm.

Fig. 7 shows the distribution of droplets diameter measured after transit through the coalescence channel. While it was possible to measure WPI-stabilized droplets and droplets stabilized by 50/50 and 75/25 (WPI/FPI) mixtures, droplets prepared by FPI alone or low WPI/FPI ratio (25/75) were highly unstable, forming large, non-spherical oil clusters. Thus, their equivalent diameter and shape eccentricity values could not be measured in observation zone 2. It was therefore concluded that although droplets could form at 0.1 mg/mL FPI, a higher concentration of these proteins is required to obtain stable droplets. Moreover, the inability to generate stable droplets in the microfluidic device when using FPI alone reflects its weaker interfacial network formation at short timescales. Unlike WPI, which instead appears to rapidly form a viscoelastic interfacial film that stabilizes droplets upon formation under the microfluidic experimental conditions (i.e., under flow and on a millisecond scale). The polydisperse composition of the FPI and lower surface activity of some of the FPI components, which may not pack as efficiently at the interface, may also play a role in reducing resistance to coalescence. These findings emphasize the necessity of WPI in mixed systems to achieve a stable, viscoelastic interface capable of preventing coalescence, even at short timescales.

WPI-stabilized droplets exhibited very low short-term coalescence, with a stability ratio (SR) of 98 %, indicating that only a minimal number of droplets coalesced as they exited the coalescence channel. With a decrease in the WPI content in the mixed systems, coalescence increased; the 75/25 WPI/FPI system showed an SR of 15 %, while the 50/50 system had an SR of 4 %. For the 25/75 WPI/FPI system and the single FPI system, the SR dropped to less than 1 % and 0 %, respectively, suggesting that these systems were highly unstable against short-term coalescence under the given conditions. Calculation of droplet volumes based on the average equivalent diameter revealed that coalesced droplets had volumes approximately twice that of the initial droplets, illustrating that coalesced droplets resulted from the merging of two smaller droplets. Shape eccentricity analysis in observation zone 2 (data not shown) indicated that all samples exhibited a decrease in shape eccentricity, with mean values around or slightly below 0.1, indicating a more spherical structure. Notably, this reduction occurred even though coalescence led to larger droplets, which would typically be more susceptible to deformation. This suggests that longer transit times, interfacial rearrangements, and the formation of a more cohesive protein film contributed to the development of a viscoelastic interfacial network, which in turn resisted droplet deformation and enabled even larger droplets to maintain a more spherical shape.

The high droplet stability of the 100 % WPI-stabilized droplets underscores the pivotal role WPI plays in the formation of a viscoelastic network at the interface, and how important this is in reducing droplet coalescence. FPI disrupted the WPI network even at low ratios, causing instability against coalescence. The relationship between interfacial viscoelasticity and the long-term stability of bulk emulsion droplets is well-documented in the literature (Grasberger et al., 2023; Hinderink, Sagis, et al., 2021) and also measured at comparable time scales (Hinderink et al., 2025; Hinderink, de Ruiter et al., 2021). The present experimental setup therefore allowed not only the characterization of interfacial behavior and protein competition but also provided insights into how different protein networks influence stability immediately after droplet formation. While the viscoelastic properties measured after 3 h, via drop tensiometry, reflect the behavior of fully developed interfacial films, the consistent trends observed between viscoelasticity and initial coalescence behavior, measured via microfluidics, suggest that proteins capable of forming cohesive viscoelastic networks at equilibrium may also contribute to interfacial resistance to coalescence during early stages of droplet formation. Nevertheless, it should be noted that interfacial rearrangements during the short timescales of droplet generation occur well before the full development of interfacial viscoelasticity, and the relationship between these timescales merits further investigation.

## 4 Conclusion

The present study examined the emulsifying properties of whey protein isolate (WPI) and faba bean protein isolate (FPI), both individually and in mixed systems, using an integrated approach combining *in situ* microfluidic-based analysis with drop tensiometry. Emulsion droplets stabilized by low concentrations of WPI exhibited improved properties, such as smaller initial droplet size and greater resistance to short-term coalescence, compared to those stabilized by FPI alone. Drop tensiometry revealed that all WPI/FPI protein ratios effectively reduced interfacial tension at the oil-water interface, with FPI and hybrid systems showing higher adsorption and rearrangement rates than WPI alone, likely due to the higher surface hydrophobicity of FPI protein molecules. It was demonstrated that both the type of protein and protein mixing significantly influenced droplet size, size distribution, shape eccentricity, and resistance to short-term coalescence. Short-term coalescence analysis showed that WPI-stabilized droplets had the lowest coalescence frequency, whereas droplets stabilized by FPI or certain hybrid systems exhibited higher instability. While stability generally positively correlated with the proportion of WPI, the presence of FPI also influenced resistance to coalescence. Both microfluidics and drop tensiometry experiments confirmed that protein mixing significantly altered interface formation, primarily by disrupting the coherence of the developed protein viscoelastic networks. The major difference identified in this study lies in how WPI and FPI, when combined, exhibit distinct and non-additive interfacial behaviors compared to their individual contributions, revealing that protein mixing does not simply dilute functionality, but rather alters the structural dynamics at the interface of the emulsion droplets depending on the ratio. These findings provide valuable insights into the interfacial behavior of mixed protein systems, contributing to a better understanding of how WPI-FPI interactions influence emulsion stability. Overall, the combined use of microfluidics and pendant drop tensiometry enabled the capture of both short-timescale interfacial dynamics and equilibrium properties, providing valuable insights into protein behavior across varying experimental and length scale conditions.

## CRediT author statement

Conceptualization: D.D.N., K.F.G., S.B.G., C.H.C, and M.C.; Data curation: D.D.N., K.F.G. and A.H.; Formal analysis: D.D.N., K.F.G. and A.H.; Investigation: A.H.; Methodology: D.D.N., K.F.G., S.B.G. and M.C.; Project administration: S.B.G. and M.C.; Resources: S.B.G. and M.C.; Software: D.D.N. and K.F.G.; Supervision: D.D.N., K.F.G., S.B.G. and M.C.; Validation: D.D.N., K.F.G., A.H., S.B.G. and M.C.; Visualization: D.D.N. and A.H.; Writing – original draft: D.D.N.; Writing - review & editing: D.D.N., K.F.G., S.B.G. and M.C.;

## Funding sources

The authors would like to thank the Villum Investigator fund (award number 0037759) for the financial support.

## Declaration of competing interest

The authors declare that they have no known competing financial interests or personal relationships that could have appeared to influence the work reported in this paper.

## Data Availability

Data will be made available on request.
