# Supplementary material for: Exploring whey and faba bean protein interactions at the oil-water interface: A combined drop tensiometry and microfluidicsstudy
Source: Curr Res Food Sci. 2025 Jul 28;11:101158. doi: 10.1016/j.crfs.2025.101158 (PMC12337646; doi:10.1016/j.crfs.2025.101158)
Supplement: Multimedia component 1 [file mmc1.docx]

**Supplementary Information**





**Figure S1:** Interfacial pressure isotherms of the single and mixed systems in relation to their ratio (WPI/FPI).

**

**

**Figure S2:** Elastic modulus of various protein solutions as a function of volumetric oscillations for both single and mixed (WPI/FPI) systems: WPI (blue line, circle), 75/25 (dark gray line, diamonds), 50/50 (medium gray line, stars), 25/75 (light gray line, hexagon), and FPI (green line, square). Each value represents the weighted average of two independent replicates, with error bars indicating the combined standard deviation, reflecting the overall uncertainty of the weighted average.

**

**

**Figure S3:** Loss modulus of various protein solutions as a function of volumetric oscillations for both single and mixed (WPI/FPI) systems: WPI (blue line, circle), 75/25 (dark gray line, rhombus), 50/50 (medium gray line, star-shaped polygon), 25/75 (light gray line, hexagon), and FPI (green line, square). Each value represents the weighted average of two independent replicates, with error bars indicating the combined standard deviation, reflecting the overall uncertainty of the weighted average.

**Table S1:** Post-hoc Tukey HSD results for equivalent diameter and shape eccentricity data presented at Table 3 and Table 4 for both single and mixed (WPI/FPI) systems: WPI (A), 75/25 (B), 50/50 (C), 25/75 (D), and FPI (E). Color code: red for insignificant difference, green for significant difference.

| **Equivalent Diameter** | | |
| --- | --- | --- |
| **Sample pairs** | **Tukey HSD Q statistic** | **Tukey HSD** |
| A vs B | 51.1026 | p<0.01 |
| A vs C | 38.0649 | p<0.01 |
| A vs D | 10.7760 | p<0.01 |
| A vs E | 58.8277 | p<0.01 |
| B vs C | 13.6362 | p<0.01 |
| B vs D | 38.9506 | p<0.01 |
| B vs E | 6.0722 | p<0.01 |
| C vs D | 26.0839 | p<0.01 |
| C vs E | 20.1932 | p<0.01 |
| D vs E | 46.0320 | p<0.01 |
| **Shape Eccentricity** | | |
| A vs B | 19.4759 | p<0.01 |
| A vs C | 29.5079 | p<0.01 |
| A vs D | 11.2113 | p<0.01 |
| A vs E | 12.7562 | p<0.01 |
| B vs C | 9.7805 | p<0.01 |
| B vs D | 29.9555 | p<0.01 |
| B vs E | 31.7840 | p<0.01 |
| C vs D | 39.7339 | p<0.01 |
| C vs E | 41.7386 | p<0.01 |
| D vs E | 1.3176 | p>0.01 |
